# Involvement of SPI-2-encoded SpiC in flagellum synthesis in *Salmonella enterica *serovar Typhimurium

**DOI:** 10.1186/1471-2180-9-179

**Published:** 2009-08-25

**Authors:** Kei-ichi Uchiya, Asami Sugita, Toshiaki Nikai

**Affiliations:** 1 Department of Microbiology, Faculty of Pharmacy, Meijo University, Tempaku-ku, Nagoya, Japan

## Abstract

**Background:**

SpiC encoded within *Salmonella *pathogenicity island 2 on the *Salmonella enterica *serovar Typhimurium chromosome is required for survival within macrophages and systemic infection in mice. Additionally, SpiC contributes to *Salmonella*-induced activation of the signal transduction pathways in macrophages by affecting the expression of FliC, a component of flagella filaments. Here, we show the contribution of SpiC in flagellum synthesis.

**Results:**

Quantitative RT-PCR shows that the expression levels of the class 3 *fliD *and *motA *genes that encode for the flagella cap and motor torque proteins, respectively, were lower for a *spiC *mutant strain than for the wild-type *Salmonella*. Further, this mutant had lower expression levels of the class 2 genes including the *fliA *gene encoding the flagellar-specific alternative sigma factor. We also found differences in flagella assembly between the wild-type strain and the *spiC *mutant. Many flagella filaments were observed on the bacterial surface of the wild-type strain, whereas the *spiC *mutant had only few flagella. The absence of *spiC *led to reduced expression of the FlhD protein, which functions as the master regulator in flagella gene expression, although no significant difference at the transcription level of the *flhDC *operon was observed between the wild-type strain and the *spiC *mutant.

**Conclusion:**

The data show that SpiC is involved in flagella assembly by affecting the post-transcription expression of *flhDC*.

## Background

Salmonellae are gram-negative bacteria causing a variety of disease syndromes in humans and animals. For example, *Salmonella enterica *serovar Typhi causes a systemic disease in human known as typhoid fever, whereas *S. enterica *serovar Typhimurium is responsible for gastroenteritis in humans and a systemic disease in mice similar to human typhoid fever. The ability of Salmonellae to survive within macrophages is required for systemic disease [1]. Important virulence factors are introduced into the host environment including the host cell cytosol using two different type III secretion systems (TTSSs) encoded on the *Salmonella *pathogenicity islands, SPI-1 and SPI-2 [2]. SPI-1 TTSS mediates bacterial entry into non-phagocytic cells [3] and SPI-2 TTSS is required for survival and replication in the intracellular environment of host cells and contributes to systemic infection in animals [4-6].

The *spiC *gene is adjacent to *spiR *(*ssrA*)/*ssrB*, a two-component regulatory gene, and is the initial gene for the operons encoding the structural and secretory components of SPI-2 [4]. Previous studies show that a strain carrying a mutation in the *spiC *gene is unable to survive within macrophages and has greatly reduced virulence in mice. The SpiC protein is necessary to inhibit the fusion of *Salmonella*-containing phagosomes with endosomal and lysosomal compartments [7]. SpiC is translocated by SPI-2 TTSS to the cytosol of the macrophages where it interacts with host proteins, *i.e*. TassC [8] or Hook3 [9], to alter intracellular trafficking. Further, several investigators report that SpiC is required for the translocation of SPI-2 effector proteins into the target cells by interacting with SsaM, a SPI-2 encoded protein [10-12]. In addition to these reports, we have shown that SpiC contributes to *Salmonella*-induced activation of the signal transduction pathways in macrophages, leading to the production of mediators such as interleukin-10, prostaglandin E_2_, and the expression of the suppressor in cytokine signaling 3 (SOCS-3) that are thought to have important roles in *Salmonella *virulence [13-15]. Additionally, our recent study shows that SpiC is involved in the expression of FliC, a component of the flagella filaments, where FliC plays a significant role in SpiC-dependent activation of the signal transduction pathways in macrophages following *Salmonella *infection [16]. However, the mechanism of how SpiC affects the expression of FliC remains unknown.

The flagellum is essential for bacterial motility. Its structure consists of a basal body, a hook, and a filament. In *Salmonella*, synthesis of the flagellum involves over 50 genes. The expression of these genes is organized into three hierarchies. At the top hierarchy is the class 1 *flhDC *operon and it is essential for transcription of all of the genes for the flagellar cascade. *flhDC *expression is influenced at the transcription or post-transcription level by a number of global regulatory factors. The class 2 operons contain genes encoding the hook-basal body-associated proteins, a few regulatory proteins, and a component of the flagellum-specific type III export pathway. The class 3 operons contain genes involved in filament formation, flagella rotation and chemotaxis [17,18]. Flagellin, a component of the filament, is transported from the cytoplasm using the flagellum-specific type III export system in the basal body where it is polymerized with the help of the cap protein FliD [19,20]. This results in the assembly of the long helical flagella filaments. *S*. *enterica *serovar Typhimurium expresses two antigenically distinct flagellins encoded by the *fliC *and *fljB *genes and are coordinately expressed using a phase-variation mechanism [17].

FliC also has a role as a potent stimulator of the immune and pro-inflammatory responses [21,22]. Several reports show that FliC activates the signal transduction pathways via Toll-like receptor 5 (TLR5) in cultured cells (*e.g*. epithelial cells) leading to the induction of immune and pro-inflammatory genes [23-26]. In addition to TLR5, flagellin was recently shown to be recognized in the host cell cytosol by two different Nod (nucleotide-binding oligomerization domain)-like receptors, Ipaf and Naip5 (also known as Birc1e) [27,28].

Here, we investigate the mechanism of how SpiC regulates flagellum synthesis in *S. enterica *serovar Typhimurium. We found that SpiC is involved in flagella assembly by affecting the post-transcription expression of the *flhDC *operon.

## Results and discussion

### Transcription of the *spiC *gene is induced during the post-exponential phase of bacterial growth in LB medium

**Figure 1:**
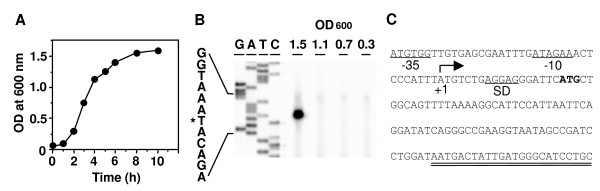
**Expression of the *spiC *gene in LB**. (A) Growth curve of wild-type *Salmonella*. An overnight culture in LB was inoculated into fresh LB at a 1:100 dilution. The cultures were grown at 37°C with aeration and monitored by measuring turbidity at an OD_600_. (B) Primer extension analysis of *spiC *transcription in LB. Bacteria were cultured in LB, and the total RNA was isolated when the OD_600 _reached 0.3, 0.7, 1.1 and 1.5. Fifty micrograms of RNA was hybridized with a 5'-end-labelled DNA fragment specific for the *spiC *gene and subjected to 6% polyacrylamide-7 M urea gel electrophoresis. The GATC lane corresponds to dideoxy chain termination sequence reactions in the region encompassing the *spiC *promoter. A single extension product was seen only at an OD_600 _of 1.5 corresponding to the stationary phase of growth. The asterisk indicates the transcription initiation site. (C) Nucleotide sequence of the *spiC *promoter region. The transcriptional start site for *spiC *is numbered as +1, and the hooked arrow indicates the direction of transcription. The proposed -10, -35, and Shine-Dalgarno (SD) sequences are underlined. The start codon is marked in bold. The double underline indicates the sequence of the designed primer for primer extension analysis.

The *spiC *gene is adjacent to the *spiR *(*ssrA*)/*ssrB *gene set and is the initial gene for the operons encoding the structural and secretory components of SPI-2 [4]. Using primer extension analysis, we first examined the expression of the *spiC *gene in bacteria grown in LB because expression of SPI-2-encoded genes has been shown to be efficiently induced under limiting conditions such as in medium containing low concentrations of Mg^2+ ^or Ca^2+ ^[29,30]. The bacteria were grown in LB, and the total RNA was isolated when the bacterial culture had an optical density at 600 nm (OD_600_) of 0.3, 0.7, 1.1, and 1.5 (Fig. 1A). As shown in Fig. 1B, the extension product was only seen when the OD_600 _was 1.5, indicating that the *spiC *gene is expressed in the stationary phase of growth.

At the same time, we determined the transcription start site for *spiC *using a primer extension analysis (Fig. 1C). The size of the extension product showed that the transcription start site of *spiC* is an adenine that lies 18 nucleotides upstream of the *spiC *initiation codon (ATG) in agreement with the result of Walthers *et al *[31]. This indicates that the SpiC protein consists of 127 amino acids with a predicted molecular mass of 14.7 kDa.

### Effect of the *spiC *mutation on the expression of class 3 flagellar genes

In a previous study, proteomic analysis using matrix-assisted desorption/ionization-time of flight mass spectroscopy showed that the level of the FliC protein, a component of the flagella filaments, was lower in the culture supernatant of a *spiC *mutant, which carries a non-polar mutation in the *spiC *gene, than in the supernatant from wild-type *Salmonella*. Further, SpiC is involved in the expression of the *fliC *gene at the transcription level [16]. These results suggest the possibility that SpiC participates in flagellar phase variation or the *fliC *gene expression directly. However, in addition to the FliC protein, we newly identified a FliD flagella protein that was decreased in the *spiC *mutant using proteomic analysis with liquid chromatography-tandem mass spectrometry (K. Uchiya, unpublished result). Taken together, these results suggest that SpiC contributes to the flagellar system by mechanisms other than phase variation or direct expression of the *fliC *gene in *S. enterica *serovar Typhimurium.

Flagella expression in *S*. *enterica *serovar Typhimurium is controlled in a hierarchical manner. At the top of the hierarchy is the class 1 *flhDC *operon that is essential for transcription of all of the genes in the flagellar cascade. The class 2 operons contain the genes encoding the hook-basal body-associated proteins, a few regulatory proteins, and a component of the type III export pathway. The class 3 operons contain genes involved in filament formation, flagella rotation and chemotaxis [17,18].

**Figure 2:**
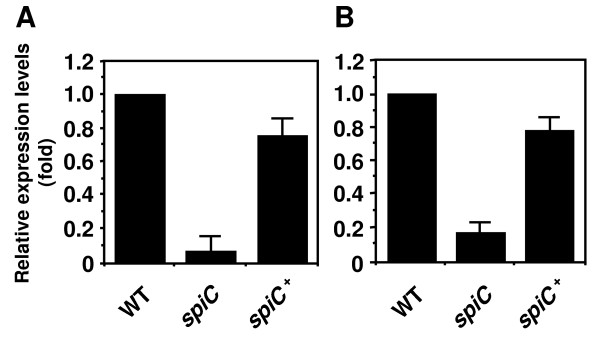
**Expression of the class 3 *fliD *and *motA *genes in the *spiC *mutant**. Bacteria were cultured in LB to an OD_600 _of 1.6, and the total RNA was extracted from the wild-type *Salmonella *(WT), *spiC *mutant strain, or *spiC *mutant strain carrying the *spiC *gene-containing plasmid pEG9127 (*spiC*^+^). Quantitative RT-PCR was conducted using a TaqMan probe. Levels of *fliD *(A) or *motA *(B) mRNA were normalized to 16S rRNA levels, and the results are shown relative to the expression in the wild-type strain. The expression levels of both genes in the *spiC *mutant were greatly reduced compared to the wild-type strain.

As described above, proteomic analysis showed that the *spiC *mutant had lower expression levels of FliC and FliD proteins, suggesting that SpiC is involved in the expression of the class 3 flagellar genes. Therefore, we first investigated the effect of the *spiC *mutation on the expression of the class 3 genes. The total RNA was isolated from bacteria grown to an OD_600 _of 1.6 in LB to induce the expression of the *spiC *gene (Fig. 1B). We analyzed the transcript levels of the *fliD *and *motA *genes that encode the flagella cap and motor torque proteins [17], respectively, using quantitative real-time PCR (RT-PCR). The transcript levels of the *fliD *and *motA *genes in the *spiC *mutant were reduced by approximately 15-fold and 6-fold compared to the wild-type strain, respectively (Fig. 2). Complementation of the *spiC *mutant with a plasmid carrying the wild-type *spiC *gene (pEG9127) restored the *fliD *and *motA *transcripts to about 80% of the level of the wild-type strain. Further, to confirm the contribution of SpiC in the regulation of class 3 flagellar gene transcription, we constructed newly a deletion mutant of the *spiC *gene using the lambda Red mutagenesis technique and examined the *motA *mRNA level. The deletion mutant showed the same phenotype as the *spiC *mutant (EG10128) used in this study (data not shown). These data indicate that SpiC has an influence on the flagellar system.

### The *spiC *mutant is defective in flagella filament formation

**Figure 3:**
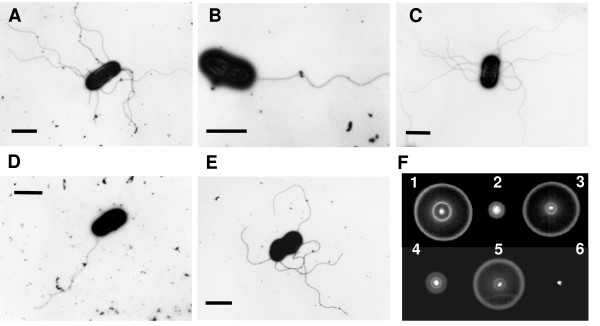
**Transmission electron micrographs and motility assays of wild-type *Salmonella *and mutant *Salmonella *strains**. A, wild-type *Salmonella*; B, *spiC *mutant strain; C, *spiC *mutant strain carrying pEG9127; D, *spiR *mutant strain; and E, *ssaV *mutant strain. The *spiC *mutant had no flagella or only a single flagellum, and the defective formation of flagella filaments in the *spiC *mutant could be restored to the wild-type phenotype by introducing pEG9127 into the *spiC *mutant. Bars represent 2 μm. (F) Motility assay of the wild-type *Salmonella *and mutant *Salmonella *strains. 1, wild-type *Salmonella*; 2, *spiC *mutant strain; 3, *spiC *mutant strain carrying pEG9127; 4, *spiR *mutant strain; 5, *ssaV *mutant strain; and 6, *flhD *mutant strain. Bacteria were inoculated on a semisolid LB plate containing 0.25% agar and incubated for 5 h at 37°C. The wild-type strain, the complemented *spiC *mutant, and the *ssaV *mutant made large swarming rings, but the *spiC *and *spiR *mutants had weak swarming abilities.

Because the flagella filament is made from the flagellin proteins FliC and FljB, we examined flagella of the respective *Salmonella *strains using electron microscopy. We found differences between the wild-type strain and the *spiC *mutant. Many flagella filaments were observed on the bacterial surface of the wild-type strain (Fig. 3A), whereas the *spiC *mutant had few flagella (Fig. 3B). Additionally, the defective flagella filament formation in the *spiC *mutant was rescued by introducing pEG9127 (Fig. 3C). The data suggest that SpiC affects the formation of flagella filaments by controlling the expression of flagellar genes. We next examined the involvement of other SPI-2-encoded virulence factors in flagella assembly. As expected, a mutation in the *spiR *gene [4], a two-component regulatory gene involved in the expression of SPI-2-encoded genes, resulted in the defective formation of flagella filaments, similar to the *spiC *mutant (Fig. 3D); however, the defective phenotype was not seen in the *ssaV *mutant that lacks a putative component of the SPI-2 TTSS (Fig. 3E) [32]. This suggests the specific involvement of SpiC in the assembly of flagella filaments. Further, we examined the effect of SpiC on formation of flagella filaments using N-minimal medium containing low Mg^2+ ^(pH 5.8) that is effective in inducing SPI-2 gene expression [29]. However, we did not observe flagella even in the wild-type strain (data not shown).

Because the absence of SpiC leads to the reduction of class 3 genes expression including the *motA *gene, which is necessary for motor rotation, we next investigated the motility of the respective *Salmonella *strains using LB semisolid plates (Fig. 3F). Like the results for flagella formation, the wild-type strain, the *ssaV *mutant, and the *spiC *mutant carrying pEG9127 made large swarming rings, whereas the *spiC *and *spiR *mutant had weak swarming abilities. And the *flhD *mutant was non-motile.

### Expression of class 2 flagellar genes in the *spiC *mutant

To examine the mechanism by which SpiC is involved in the expression of the class 3 genes, we focused on the class 2 *fliA *gene encoding the flagellar-specific alternative sigma factor σ^28^, which is required for transcription of the class 3 promoters [33,34]. The activity of the transcription factor σ^28 ^is negatively regulated by direct interaction with an anti-σ^28 ^factor, the FlgM in the cell [35,36]. FlgM is excreted out of the cell through the flagellum-specific type III export apparatus, leading to the induction of *fliA *gene transcription [37-39]. SpiC is reported to be required for secretion of some virulence factors from the cytoplasm using the SPI-2 TTSS [10,11], although the molecular mechanism is not known. Several genes encoding the SPI-2 TTSS and the flagellum-specific type III export system show sequence similarities [18,40]. Therefore, in addition to its role in SPI-2 TTSS, SpiC might participate in the export of FlgM proteins from the cytoplasm via the type III flagellar protein export system. To examine this possibility, cell lysates were prepared and the level of intracellular FlgM was assessed using Western blot with anti-FlgM antibody. Western blot analysis showed that the level of FlgM in the wild-type cell was higher than that in the *spiC *mutant (data not shown), indicating that a decrease in class 3 genes expression in the *spiC *mutant is due to an FlgM-independent mechanism.

**Figure 4:**
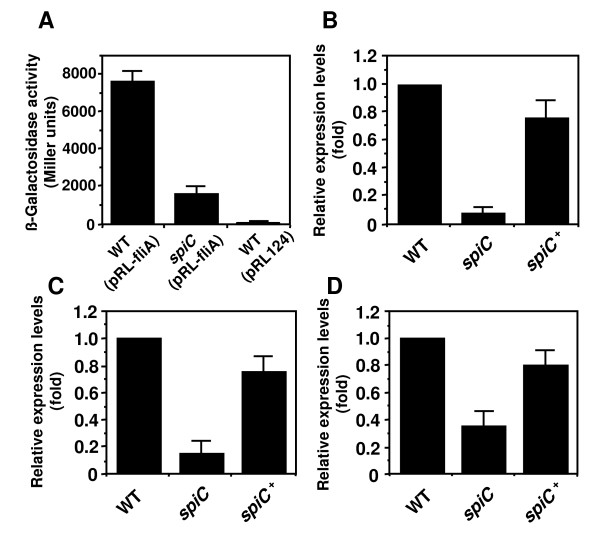
**Expression of the class 2 genes in the *spiC *mutant**. (A) β-galactosidase activity from *fliA-lacZ *transcription fusion expressed by wild-type *Salmonella *(WT) and *spiC *mutant strain grown in LB to an OD_600 _of 1.6. β-galactosidase activity is expressed in Miller units. WT (pRL124) carries the vector with the promoterless *lacZ*. Quantitative analysis of *fliA *(B), *flgB *(C), or *fliF *(D) mRNA expression. Bacteria were cultured in LB, and the total RNA was extracted from the wild-type *Salmonella*, *spiC *mutant strain, or *spiC *mutant strain carrying pEG9127 (*spiC*^+^) when the OD_600 _was 1.6. Quantitative RT-PCR was conducted using a TaqMan probe. Levels of each mRNA were normalized to the 16S rRNA concentration, and the results are shown relative to the expression in the wild-type strain. The expression levels of the *fliA, flgB*, or *fliF *gene in the *spiC *mutant were greatly reduced compared to the wild-type strain.

In subsequent studies, we measured the expression level of the *fliA *gene by fusing the transcription regulatory region of *fliA *to *lacZ *in pRL124, as described in the Materials and Methods (Fig. 4A), and quantitatively measured the expression level using RT-PCR (Fig. 4B). The expression level of the *fliA *gene in the *spiC *mutant was greatly reduced compared to the wild-type strain. In addition to the *fliA *gene, we further investigated the influence of SpiC on the expression of the class 2 *flgB *and *fliF *genes [17]. As shown in Fig. 4C and 4D, quantitative RT-PCR analysis showed that the transcript levels of the *flgB *and *fliF *genes in the *spiC *mutant were reduced approximately 7-fold and 3-fold in comparison to the wild-type strain, respectively. These results indicate that SpiC affects the regulation of class 2 genes transcription, and suggest the involvement of SpiC in the expression of the class 1 *flhDC *gene, which functions as the master regulator in flagellar genes expression [17].

### SpiC is required for the post-transcriptional expression of the master regulator, FlhDC

The class 1 genes products FlhD and FlhC form a heterotetramer that activates the σ^70 ^promoter in the class 2 genes by interacting with the RNA polymerase α subunit [41,42]. *flhDC *expression is influenced at the transcription or post-transcription level by a number of global regulatory factors. For example, cyclic AMP-CRP [43-46], H-NS [46,47], QseBC [48], CsrA [49], and the heat shock-induced chaperones, DnaK, DnaJ, and GrpE [50], function as positive regulators, while negative regulation is mediated by OmpR [51], RcsCDB [52], LrhA [53], and ClpXP [54].

**Figure 5:**
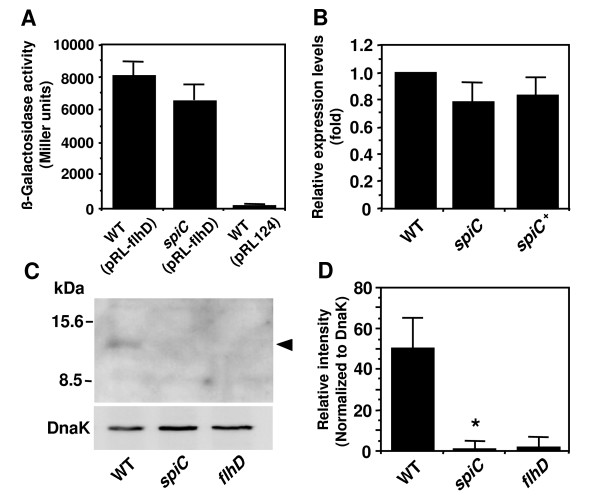
**Effect of the *spiC *mutation on *flhDC *expression**. (A) β-galactosidase activity from *flhD-lacZ *transcriptional fusion expressed by wild-type *Salmonella *(WT) and the *spiC *mutant strain grown in LB to an OD_600 _of 1.6. β-galactosidase activity is expressed in Miller units. WT (pRL124) carries the vector with the promoterless *lacZ*. (B) Quantitative analysis of *flhD *mRNA expression. Bacteria were cultured in LB, and the total RNA was extracted from the wild-type *Salmonella *(WT), *spiC *mutant strain, or *spiC *mutant strain carrying pEG9127 (*spiC*^+^) when the culture OD_600 _was 1.6. Levels of *flhD *mRNA were normalized to the 16S rRNA concentration, and the results are shown relative to the expression in the wild-type strain. In both assays, no significant difference in the expression levels of the *flhD *gene was observed between the wild-type strain and the *spiC *mutant. (C) Western blot analysis of FlhD expression. Whole-cell lysates from the wild-type *Salmonella *(WT), *spiC *mutant strain, or *flhD *mutant strain were prepared and were analyzed using Western blot with an anti-FlhD peptide antibody or an anti-DnaK specific antibody. The black arrowhead indicates FlhD protein. Molecular masses are indicated on the left. (D) Densitometric analysis of the amount of FlhD normalized to the amount of DnaK, a bacterial heat shock protein, in the same samples. The *spiC *mutant showed a reduced expression level in FlhD protein compared to the wild-type strain. *P < 0.001, significantly different from the wild-type strain.

Because SpiC was found to affect the expression of the class 2 genes including the *fliA *gene, we examined the involvement of SpiC in the *flhDC *operon expression using an *flhDC-lacZ *fusion (Fig. 5A), and measured the level using quantitative RT-PCR (Fig. 5B). Although the *spiC *mutant showed a slight reduction in *flhD *expression compared to the wild-type strain, no significant difference in the *flhD *expression level was observed between the wild-type strain and the *spiC *mutant. Reports show that the *flhD *expression level is reduced approximately 2- to 3-fold by mutation to the regulatory genes affecting the *flhD *expression at the transcription level [46,48,51,53]. Together with these findings, we concluded that the reduced level of the class 2 gene expression in the *spiC *mutant is not dependent on *flhDC *transcription. To investigate whether SpiC participates in *flhD *expression at the post-transcription level, we performed Western blot analysis with anti-FlhD peptide antibody. Although the detection level of FlhD was low, we found significant differences between the wild-type strain and the *spiC *mutant (Fig. 5C and 5D). The absence of *spiC *led to the reduced expression of the FlhD protein, indicating that SpiC is involved in *flhD *expression at the post-transcription level.

Although the molecular mechanism by which SpiC contributes to the post-transcription regulation of the *flhD *expression remains unknown, it is thought that SpiC directly or indirectly participates in either *flhD *translation or in the stability of the FlhD protein. Almost all of the positive regulators that involved in *flhDC *expression regulate their expression at the transcription level [45-47,50], while CsrA, a RNA-binding protein, stimulates *flhDC *expression using a post-transcription mechanism [49]. CsrA is thought to allow *flhDC *translation by binding to the 5' segment of the *flhDC *mRNA and stabilizing its mRNA. The Csr system consists of CsrA and the two small regulatory RNAs, *csrB *and *csrC*. The activity of CsrA is reported to be antagonized by *csrB *and *csrC *RNAs [55] where gene expression is controlled by the BarA/SirA two-component regulatory system that is involved in the expression of SPI-1-encoded genes [56-58]. One hypothesis is that SpiC affects FlhDC expression via a Csr post-transcription regulatory system. Therefore, we investigated the effect of SpiC on *csrB *and *csrC *expression using quantitative RT-PCR. However, no differences in the expression levels of these genes were observed between the wild-type strain and the *spiC *mutant (data not shown). More research is required to clarify the molecular mechanism in how SpiC regulates the post-transcriptional expression of the *flhDC*.

We next examined the expression of FlhD at bacterial growth phase of OD_600 _of 0.7 in LB, because the *spiC *expression is induced at over an OD_600 _of 1.5 when the bacteria are grown in LB. However, the expression level of FlhD in the *spiC *mutant was reduced compared to the wild-type strain even in the exponential growth phase (data not shown), indicating that the FlhD expression is not strictly growth phase-dependent. We cannot explain this phenomenon until the mechanism by which SpiC regulates the post-transcriptional expression of the *flhDC *or the molecular mechanism of SpiC become clear.

As described above, the BarA/SirA system is involved in not only the flagella gene expression but also the SPI-1 gene expression. Phosphorylated SirA directly interacts with promoters of the *hilA *and *hilC *genes that are the SPI-1-encoded transcription regulator genes [58]. HilA, a member of the OmpR/ToxR family, directly activates transcription of the *inv/spa *and *prg/org *promoters on SPI-1 [59]. In addition to the BarA/SirA system, the AraC-like regulator RitA directly controls the *hilA *expression leading to SPI-1 gene expression, while RitB, a helix-turn-helix DNA binding protein, negatively regulates the expression of the *flhDC *[60]. Reports also show that the ATP-dependent ClpXP protease negatively regulates the expression of flagella and SPI-1 gene [54,61]. Interestingly, mutation in the SPI-2 genes also affects the expression of the SPI-1 gene [62]. And thus many reports show the relationship of flagella synthesis and SPI-1 gene expression.

Our recent studies show that the SpiC-dependent expression of FliC plays a significant role in activation of the signaling pathways leading to the induction of SOCS-3, which is involved in the inhibition of cytokine signaling, in *Salmonella*-infected macrophages [16]. Lyons *et al*. [63] also reported that infection of polarized epithelial cells by *Salmonella *leads to IL-8 expression by causing the SPI-2-dependent translocation of flagellin to a basolateral membrane domain expressing TLR5. Together with our previous results, these findings suggest the involvement of FliC in SPI-2-dependent events in the pathogenesis of *Salmonella *infection.

## Conclusion

In conclusion, here we show that SpiC encoded within SPI-2 is required for flagella assembly in *S. enterica *serovar Typhimurium. We concluded that the mechanism is due to the involvement of SpiC in the post-transcriptional expression of FlhDC. The data indicate the possibility that SPI-2 plays a role in *Salmonella *virulence by making use of the flagellar system.

## Methods

### Bacterial strains, plasmids, and growth conditions

The bacterial strains used in this study were derived from the wild-type *S. enterica *serovar Typhimurium strain 14028s. The *spiC*::*kan *derivative EG10128 was described by Uchiya *et al*. [7]. The deletion mutant in the *flhD *gene was constructed using the Red recombination system [64]. To delete the *flhD *or *spiC *gene, a kanamycin resistance gene flanked by FLP recognition target sites from plasmid pKD4 was amplified using PCR with primer regions homologous to the *flhD *gene (5'-TGCGGCTACGTCGCACAAAAATAAAGTTGGTTATTCTGGATGGGAGTGTAGGCTGGAGCTGCTTC-3' and 5'-CGCGAGCTTCCTGAACAATGCTTTTTTCACTCATTATCATGCCCTCATATGAATATCCTCCTTAGT-3') or the *spiC *gene (5'-TTGTGAGCGAATTTGATAGAAACTCCCATTTATGTCTGAGGAGGGGTGTAGGCTGGAGCTGCTTC-3' and 5'-AGATTAAACGTTTATTTACTACCATTTTATACCCCACCCGAATAACATATGAATATCCTCCTTAGT-3'). Kanamycin-resistant strains were obtained by transforming the PCR products into strain 14028s harboring λ Red recombinase on the plasmid pKD46. Disruption of the *flhD *or *spiC *gene was confirmed using PCR with *flhD *or *spiC *gene-specific primers. The kanamycin resistance gene was then removed by transforming the strain with plasmid pCP20 that expresses FLP recombinase, resulting in an in-frame deletion of the *flhD *or *spiC *gene. Plasmid pEG9127 is a derivative of pBAC108L containing the cloned *spiC *gene [7]. The bacteria were grown at 37°C in Luria broth (LB). Kanamycin was used at 50 μg/ml.

### RNA preparation and primer extension analysis

Bacteria were grown in LB. When the OD_600 _reached 0.3, 0.7, 1.1, and 1.5, the total RNA was isolated using an RNeasy kit (Qiagen, Hilden, Germany) in accordance with the manufacturer's protocol. The RNA (50 μg) was mixed with ^32^P-end-labeled synthetic oligonucleotide (5'-GCAGGATGCCCATCAATAGTCATT-3'), and 50 units of SuperScript II reverse transcriptase (Invitrogen, Carlsbad, CA) was added to 30-μl reaction mixtures containing 1 mM of deoxynucleoide triphosphates, 5 mM dithiothreitol, and 1 unit of RNasin/μl. The reaction was performed at 42°C for 1 h. The extension products were analyzed using electrophoresis on a 6% polyacrylamide-7 M urea gel and compared to sequence ladders initiated with the same primer.

### Quantitative RT-PCR

Bacteria were grown in LB, and the total RNA was isolated when the OD_600 _reached 1.6. The isolated RNA was treated with DNase I (Invitrogen) to remove contaminating DNA, and 2 μg of RNA was reverse-transcribed using SuperScript II reverse transcriptase with random primers. Real-time PCRs were performed in a 50-μl reaction mixture containing 1 μl cDNA, 0.9 μM each primer, 0.25 μM each fluorescent probe, and TaqMan Universal Master Mix (Applied Biosystems, Foster City, CA). Amplification was performed in 96-well optical plates using the 7300 Real-Time PCR System (Applied Biosystems) with an initial incubation of 2 min at 50°C; followed by 10 min at 95°C; and then 40 cycles: 95°C for 15 s and 60°C for 1 min. The housekeeping gene 16S ribosomal RNA (rRNA) was used as an internal standard for quantification of the total RNA. The primer pairs and fluorescent probes were designed using Primer Express Software ver. 3.0 and were synthesized by Applied Biosystems. The specific fluorescent probes were labeled at the 5'-end with the reporter dye 6-carboxyfluorescein (FAM). The sequences of the primer-probe combinations are shown in Table 1. Threshold cycle values were calculated from the amplification plots, and the amount of each gene expression was determined relative to the level of the gene expression in wild-type *Salmonella *after both values were normalized to the 16S rRNA levels. Each sample was analyzed in triplicate.

**Table 1:** Oligonucleotide primers and fluorescent probes used in RT-PCR

Target gene	Nucleotide sequence (5'-3')
*fliD*	CGCGAAGCTGAACGTAAACG
	GCGTCGGTTACGGTATTGC
	*6-FAM-TCTGACGCTCAATGTC*
*motA*	GCTTTCATGGGTTTCAATCTCTTCA
	CAGCGGCAACATGAATACGTT
	*6-FAM-TCAACGCTTCAATTTC*
*fliA*	TCGATGCTATCGCCATGCT
	TTGCGGAGTATCGTCAGATGTT
	*6-FAM-CGCCACTCATCGTAAGA*
*fliF*	GCAGACGGAAGAGCACTACAG
	GCCTACCTGTTCGCTAATATTCAAC
	*6-FAM-TCGAAGGCCACTCTGC*
*flgB*	CGTCGCGTTAACGTTGACTTC
	TCCACTGCGGGAGAAGAGA
	*6-FAM-CTCTCACCATATTCCC*
*flhD*	TGATGATCGTCAAACCGGAAA
	TGCCGCAGATGGTCAAACTG
	*6-FAM-AACTAACTGGTTCGTCTCC*
16S rRNA	AGATGGGATTAGCTTGTTGGTGA
	GTAACGTCAATGCTGCGGTTA
	*6-FAM-CCACAACACCTTCCTC*

Fluorescent probes are shown in italic.

### Preparation of whole-cell proteins

An overnight culture in LB was inoculated into 15 ml of fresh LB at a 1:100 dilution. The cultures were grown at 37°C with mild aeration to an OD_600 _of 1.6 (the *spiC*-inducing condition). After a 1-ml sample of the culture was centrifuged at 18,500 × *g *for 15 min, the bacterial pellet suspended in 1 ml of cold water was mixed with trichloroacetic acid (final concentration 6%), placed on ice for 30 min, and centrifuged at 14,000 × *g *for 20 min. After drying, the pellets were dissolved in 100 μl of sodium dodecyl sulfate (SDS)**-**sample buffer and boiled for 5 min.

### Construction of the fliA or flhD-lacZ fusion on a plasmid

To construct the transcriptional fusion of the *fliA *or *flhD *promoter region to the promoterless *lacZ *gene using the promoter-probe vector pRL124 [65], a 0.51-kbp DNA fragment containing the *fliA *promoter region or a 0.73-kbp DNA fragment containing the *flhD *promoter region were amplified using PCR with the following primers: for *fliA*, 5'-ACGCGTCGACTATGCGCCTGTTAGGGCGCG-3' and 5'-CGGGGTACCCACCCAATCGCGGCTGCGTA-3'; and for *flhD*, 5'-ACGCGTCGACGCCACATTAATGTGAAGGAC-3' and 5'-CGGGGTACCCGGATGTATGCATTGTTCCC-3'. The PCR products digested with Sal1 and Kpn1 were ligated into the same site in pRL124, producing pRL-fliA and -flhD.

### β-Galactosidase assay

Bacteria were grown overnight in LB at 37°C and diluted to 1:100 in fresh LB and grown with aeration to an OD_600 _of 1.6. β-galactosidase activity was measured using the substrate o-nitrophenyl β-D-galactoside as described elsewhere [66]. Each sample was assayed in triplicate.

### Transmission electron microscopy

Bacterial cells grown in LB for 20 h at 37°C without shaking were deposited on carbon-film grids, partially dried, and stained with 2.0% uranyl acetate. The negatively stained samples were observed using a 2000EX electron microscope (JEOL) at an acceleration voltage of 100 kV.

### Western Blot Analysis

Whole-cell proteins (150 μg) from bacteria were fractionated in 16% Tricine-SDS-polyacrylamide gel, electrophoresed, and then electrotransferred onto a polyvinylidene difluoride membrane (Millipore, Bedford, MA) as described previously [14]. The bands were detected using the ECL plus Western blot detection system (GE Healthcare, Little Chalfont, UK) according to the manufacture's instructions. The peptide fragment, DHQTITRLTQDSRV, from the FlhD polypeptide was synthesized and an antiserum specific for the oligopeptide was obtained by immunization of rabbits with the peptide coupled to keyhole limpet hemocyanin using benzidine. The resulting anti-FlhD peptide antibody was used at a dilution of 1:300. DnaK was detected with a 1:1000 dilution of anti-DnaK antibody (Assay designs, Ann Arbor, MI). Bands were analyzed using a GS-800 calibrated densitometer (Bio-Rad).

### Statistical analysis

Each experiment was performed at least three times. The results are expressed as means ± the standard deviations. The data were analyzed using analysis of variance with the Dunnett's test. A value of *p *< 0.05 was considered statistically significant.

## Authors' contributions

AS performed experiments and analyses. TN helped to draft the manuscript. KU contributed to the experimental designs and drafted the manuscript. All authors read and approved the final manuscript.
